# Financial stress and mental health among higher education students in the UK up to 2018: rapid review of evidence

**DOI:** 10.1136/jech-2019-212154

**Published:** 2019-08-12

**Authors:** Tayla McCloud, David Bann

**Affiliations:** 1 Division of Psychiatry, University College London, London, UK; 2 Centre for Longitudinal Studies, UCL Institute of Education, London, UK

**Keywords:** Debt, financial stress, mental health, higher education

## Abstract

**Introduction:**

In the United Kingdom and many other countries, debt accrued during higher education has increased substantially in recent decades. The prevalence of common mental health problems has also increased alongside these changes. However, it is as yet unclear whether there is an association between financial stress and mental health among higher education students.

**Methods:**

We conducted a rapid review of the peer-reviewed scientific literature. Eligible studies were English-language publications testing the association between any indicator of financial stress and mental health among higher education students in the UK. Papers were located through a systematic search of PsychINFO, PubMed and Embase up to November 2018.

**Results:**

The search strategy yielded 1272 studies—9 met the inclusion criteria. A further two were identified through hand-searching. The median sample size was 408. Only three of seven studies found an association between higher debt and worse mental health. There was a consistent cross-sectional relationship between worse mental health and both experience of financial difficulties (seven of seven studies) and debt worry/financial concern (four of five studies), though longitudinal evidence was mixed and limited to six studies.

**Conclusion:**

Among higher education students in the UK, there is little evidence that the amount of debt is associated with mental health. However, more subjective measures of increased financial stress were more consistently associated with worse mental health outcomes. Nevertheless, the identified evidence was judged to be weak; further research is required to examine whether links between financial stress and mental health outcomes are robust and causal in nature.

## Introduction

The widening participation of young people in higher education in recent decades has focused attention on student mental health and well-being, and raised concerns regarding the impact of financial stress. For example, in England, tuition fees have increased and grants have been replaced by loans, leaving the average undergraduate with debts of around £50 000.^1^ In addition to future debt concerns, a sizeable fraction of higher education students in the UK (21% in 2004) also experience personal financial crises each year.^2^

There is growing concern among students, the wider public, and policy-makers in the UK that increasing financial pressures may explain co-occuring trends in worsening student mental health.^3^ A recent report comparing 2015/2016 with 2006/2007 found that the number of UK higher education students disclosing mental health problems increased fivefold, and university deaths by suicide increased by 79%.^4^ Seemingly as a result, student mental health services are under pressure, with demand in the UK increasing^3^ but not being met.^5^ Consequences of poor mental health can include academic underperformance and dropping out of university,^6 7^ and thus these trends have implications for individuals, their families, the higher education sector and the public health community.^8^

A number of studies suggest that student debt may be related to students’ mental health problems,^9 10^ but other studies have reported null findings.^7 11^ Such links may be anticipated, given the substantial literature on the relationship between debt and mental health in the general population. A 2013 systematic review^10^ found that more severe debt was associated with depression, suicide, drug and alcohol dependence and psychotic disorders. In addition to amount of debt, a 2018 survey of over 3000 UK students^12^ indicated that 3 in 5 students worried about paying back their loan, 84% worried about having enough money to live on and 50% believed that their mental health suffered as a result of financial difficulties.

Given existing evidence, it is difficult to conclude with any certainty whether financial stress is associated with students’ mental health, nor which domain of financial stress is most important—the amount of debt, experience of financial difficulties or worry about debt. Additionally, it remains to be seen whether associations are bidirectional, or robust to adjustment for potential confounders such as socioeconomic background^13^ and preceding mental health problems.^14^ As such, the present review sought to rapidly summarise the existing literature in this area, focusing on UK students.

## Methods

We conducted a rapid review of peer-reviewed scientific literature examining the links between financial stress and mental health among higher education students in the UK. Rapid reviews are a form of evidence synthesis which are typically systematic in nature but omit particular steps taken in a full systematic review (eg, only one author screens the identified papers). They can be considered a streamlined approach to traditional systematic review methods and yield valid inferences on the research topic addressed, although with less certainty than a full systematic review.^15^

Three databases (PsychINFO, PubMed and Embase) were searched for any papers up to 29 November 2018. The search term ‘student’ was combined with keywords related to mental health and finances. Owing to the scarcity of literature in the field, any studies which reported any measure of financial situation, stress or difficulties or debt, however defined, were considered to be measuring financial stress. Owing to the time constraints and the large number of studies examining ‘debt stress’ and ‘financial stress’ but not mental health outcomes, the word ‘stress’ was not included among the mental health terms. We therefore executed the following search:

(Mental or Depress* or Anxi*) AND (Debt* or Loan* or Financ*) AND Student

English-language studies were included if they measured associations between mental health and financial stress in higher education students within the UK (at any time). The authors preidentified four key studies in the field eligible for inclusion and ensured that this search strategy captured all of them. Titles and abstracts were screened by one author (TM) for eligibility, followed by the full texts of any seemingly eligible papers. Additionally, we hand-searched papers from the most recent relevant systematic review^10^ and recent work by lead authors on included studies to ensure that no key papers had been missed.

Data were then extracted by one author (TM) into a table devised by the authors; see table 1. Data extracted included year(s) of data collection, sampling method, number of participants and response rate, any relevant outcome measures, analytical strategy (including confounding adjustment) and main findings (regardless of summary measure used). We narratively summarised our findings, stratified by the indicator of financial stress since meta-analysis was not judged to be possible.

**Table 1:** Characteristics of included studies

Study	Year(s) of data collection	Sampling/selection	N and response rate	Study design	Finance measures	Mental health measures	Other outcome measures	Analytical strategy	Main findings
Andrews and Wilding, 2004^2^	2000–2002	One pre-1992 University in London, England (Royal Holloway). Undergraduates only.	676 students at time point 1 (T1), 351 at time point 2 (T2); response rate 76%	Prospective cohort—two time points. One month before first year and halfway through second year	Financial difficulties: major financial crisis, unable to afford essentials (item taken from adverse life experiences list)	Anxiety and depression (HADS), adverse life experiences (death, separation, etc)	Examination results (at the end of second year) obtained from University Registrar	Logistic regression to determine relative contribution of variables to the prediction of anxiety and depression (separately) midcourse. Adjusted for mental health at baseline, caseness of anxiety or depression midcourse, gender, age and ethnicity.	Found an association between financial difficulties and depression, but not anxiety. Depression mediated the relationship between financial difficulties and subsequent exam performance.
Cooke *et a* *l*, 2004^11^	2000–2003	One pre-1992 University in England (University of Leeds). Undergraduates only.	38% at T1 (n=2146), 23% (n=1360) at T2 and 26% (n=1391) at T3	Prospective cohort—three time points at the end of semester one each year	Anticipated amount of debt (T3 only); financial concern (one question) and debt worry (one question, T3 only)	Mental health functioning, problems and general mental health (CORE-GP)		Effect size differences using z scores (0.4 SD units as threshold).	Found an association between debt worry/financial concern and mental health, but no association between amount of debt and mental health.
Jessop *et al*, 2005^16^	Unknown	Middlesex University, London; overseas students excluded (also conducted in Finland but separate analyses reported here only). Graduate status unclear.	89 students; response rate 95.2% (across London and Finland)	Cross-sectional survey	Amount of debt; financial concern: 6 5-point Likert-scale items, for example, ‘I worry about my financial situation’	General health (SF-12)—includes questions on mental health and its interference with role		Correlations; multiple regressions with amount of debt, age, gender, financial concern, no. of hours worked and units of alcohol as predictors and each of the SF-12 dimensions as outcomes.	Found higher debt and more financial concern were correlated with worse mental health, but financial concern did not predict mental health functioning. Financial concern was positively correlated with amount of debt.
Richardson *et al*, 2015^8^	2012–2014	All UK student unions contacted (46 of 114 agreed). Undergraduates only.	390 students; response rate unclear (very low)	Prospective cohort—four time points ~2 months apart over first 2 years at university	Fee amount (amount of debt): 0-£2.9k, 3–4k or 8–9k	Anxiety (GAD-7), depression (CES-D), general mental health (CORE-GP), stress (PSS)		Correlation between tuition fee group and student well-being, with no apparent adjustment for confounders.	Found no association between debt and well-being.
Richardson *et al*, 2015^23^	Unknown	All UK student unions contacted (46 of 114 agreed). Undergraduates only.	444 students (completed baseline and at least one other time point); response rate unclear (very low)	Prospective cohort—four time points 3–4 months apart over first 2 years at university	Financial difficulties over past 6 months (IFS)	Attitudes towards food and eating (EAT-26)		Hierarchical linear multiple regressions to test whether IFS at baseline predicted later EAT-26 scores (and vice versa), after adjusting for socioeconomic status, baseline EAT-26, gender, ethnicity and age. Significant findings repeated separately for each gender. Missing data replaced with the mode.	Found that financial difficulties predict more severe long-term eating attitudes, for women only. Mixed findings for a bidirectional relationship.
Richardson *et al*, 2017^14^	2012–2014	All UK student unions contacted (46 of 114 agreed). Undergraduates only.	454 students; response rate unclear (very low)	Prospective cohort—four time points ~2 months apart over first 2 years at university	Financial difficulties: over past 6 months (IFS), considering abandoning course for financial reasons; debt stress and view of loan	Anxiety (GAD-7), depression (CES-D), general mental health (CORE-GP), stress (PSS)		Linear hierarchical multiple regression used to see whether financial variables predicted mental health scores at each time point, adjusting for age, gender, disability, mature student status, ethnicity and mental health at baseline. Regression models with baseline IFS, socioeconomic status, demographics and all mental health measures to predict IFS at T2. Missing data filled in with the mode.	Found an association between financial difficulties and mental health cross-sectionally, but mixed longitudinal findings. Evidence points to a potential bidirectional relationship. Greater subjective stress about debt predicted worse mental health cross-sectionally, and at the longest follow-up point.
Richardson *et al*, 2018^20^	2012–2014	All UK student unions contacted (46 of 114 agreed). Undergraduates only.	408 students; response rate unclear (very low)	Prospective cohort—four time points ~2 months apart over first 2 years at university	Amount of debt; financial difficulties: over past 6 months (IFS), considering abandoning course for financial reasons; debt stress	Psychosis risk (Prodromal Questionnaire-Brief Version; PQB)		Hierarchical multiple linear regression with financial variables as predictors and PQB score at baseline as outcome (controlling for gender, age and ethnicity). Significant variables entered into a regression with PQB over time as outcome.	Found that debt stress and amount of debt are not associated with psychosis risk. Some indicators of financial difficulies are associated with psychosis risk (i.e. IFS scores), but others are not (i.e.considering abandoning). No evidence of bidirectionality.
Roberts *et al*, 1998^6^	‘Over the past year’ —1997–1998 assumed.	Unknown British university. Undergraduates (83%) and postgraduates.	103 students; response rate unclear	Cross-sectional survey	Amount of debt; financial difficulties: difficulty paying bills, considering abandoning course for financial reasons	General mental health (GHQ)		Not reported	Found that there is an association between mental health and financial difficulties. Poorer mental health was significantly related to difficulty paying bills. People who had considered abandoning for financial reasons had significantly poorer mental health.
Roberts *et al*, 1999^21^	Unknown	Students from two universities in London—one pre-1992 and one post-1992. Undergraduates (90%) and postgraduates	360 students; response rate 65%	Cross-sectional survey	Amount of debt; financial difficulties: difficulty paying bills, considering abandoning for financial reasons	General mental health (SF-36 and GHQ-12)		Regression models with GHQ and SF-36 subscale scores as outcome and hours worked, difficulty paying bills and considering dropping out as predictors. Structural equation modelling of pathways linking financial variables and mental health.	Found that there is an association between mental health and financial difficulties (difficulty paying bills and considering dropping out). Conclude that there are two pathways through which amount of debt is associated with mental health.
Roberts *et al*, 2000^22^	Unknown	Students from two universities in London—one pre-1992 and one post-1992. Undergraduates (87%) and postgraduates	482 students; response rate 66%	Cross-sectional survey	Amount of debt; financial difficulties: difficulty paying bills, considering abandoning course for financial reasons	General mental health (SF-36 and GHQ-12)	Help seeking: whether have consulted a GP in past 2 weeks and satisfaction with most recent consultation	Linear regression models adjusted for age and sex to assess association between financial difficulties and GHQ scores. Regression models to examine the relationship between debt and help seeking (adjusted for age and gender). Structural equation modelling of pathways linking financial variables and mental health.	Found that there is an association between financial difficulties (difficulty paying bills and considering dropping out) and mental health. Two pathways confirmed (as above).
Ross *et al*, 2006^7^	2004	Undergraduate medical students from one university in Scotland (University of Aberdeen).	352 responses out of 900 students (39% response rate)—334 included.	Cross-sectional survey	Amount of debt; questions on worrying about money	General mental health (GHQ-12)	Examination results (students’ rankings relative to the rest of the year group)	Pearson’s partial correlations controlling for effect of year.	Found that indicating that worrying about money affects performance was associated with poorer mental health. Mixed findings on relationship between debt and mental health.

CES-D, Center for Epidemiological Studies - Depression scale; CORE-GP, General Population version of the Clinical Outcomes in Routine Evaluation Questionnaire; EAT-26, Eating Attitudes Test-26; GAD-7, 7-item Generalised Anxiety Disorder Assessment; GHQ, General Health Questionnaire; GP, General Practitioner; HADS, Hospital Anxiety and Depression Scale; IFS, Index of Financial Stress; PSS, Perceived Stress Scale; SF-12, 12-item Short Form health survey; SF-36, 36-item Short Form health survey.

## Results

### Study characteristics

**Figure 1:**
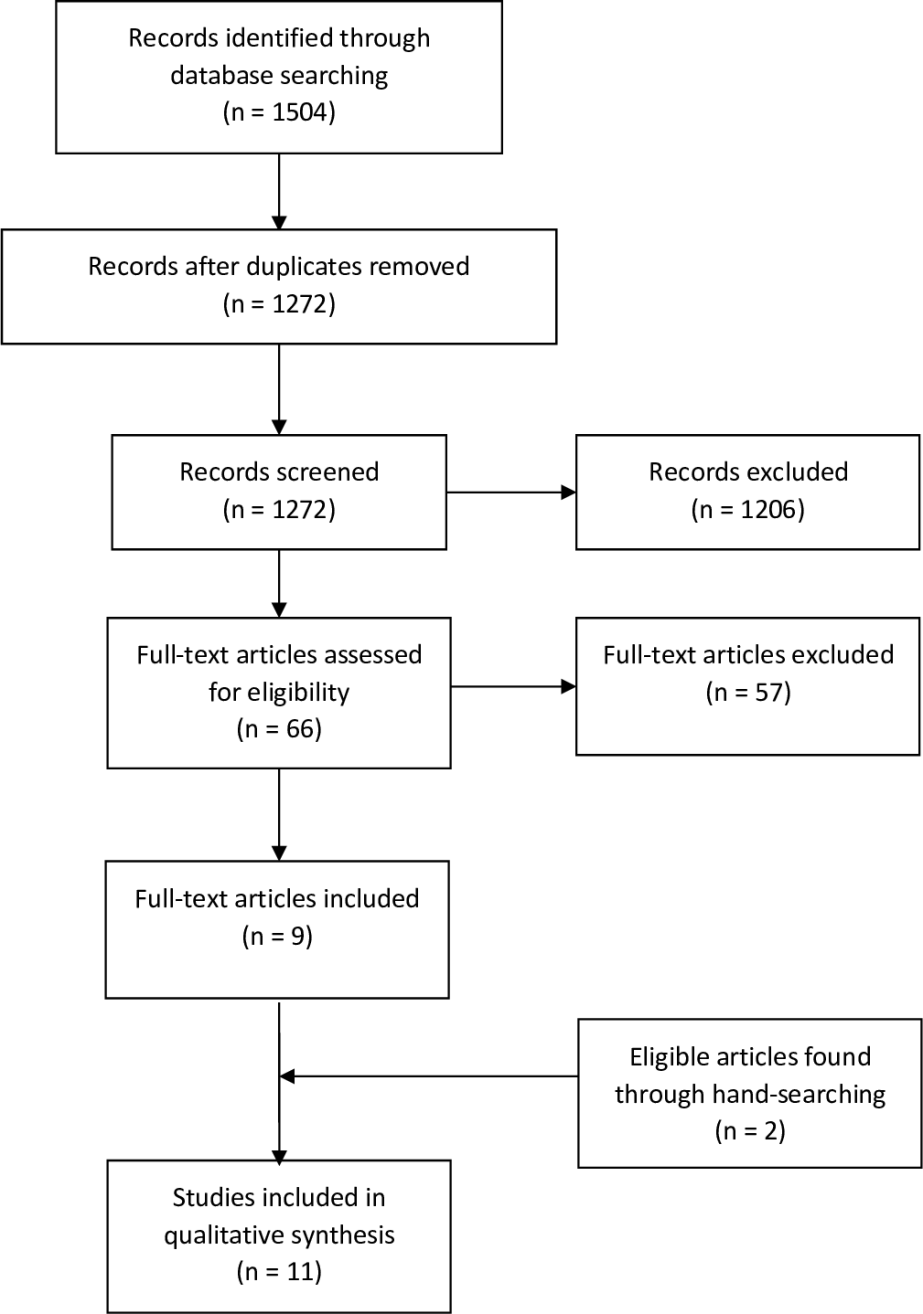
Flow diagram.

The search strategy yielded 1272 results once duplicates were excluded, and only 9 studies were deemed to meet the inclusion criteria (figure 1). A further two studies were identified through hand-searching. Thus, the final number of included studies was 11.

The earliest study took place between 1997 and 1998, and the most recent between 2012 and 2014. Seven of the studies only recruited undergraduates, three had a mixed sample of undergraduates and postgraduates with over 80% undergraduates and graduate status was unclear for one study.^16^ Four of the included papers reported contacting all UK student unions, two recruited participants from two London universities and the five remaining studies were all based at one institution only. Reported response rates ranged from 38% to 95% and were unreported for five studies. Sample sizes ranged from 89 to 2146 with a mean of 536.7 (SD 558.5).

All of the included studies were survey based. Five of the included papers reported on cross-sectional surveys, and the remaining six on data from longitudinal prospective studies. The longest study spanned 3 years (one time point per year), while the remaining five were conducted over the first 2 years of students’ degrees.

In terms of financial measures, seven studies used the amount of debt students anticipated leaving university with or their tuition fee amount. Five papers reported asking students if they had ever considered abandoning their studies due to financial issues, while four asked about other financial difficulties the students had experienced. Three papers also reported using the Index of Financial Stress^17^ (IFS), a measure of financial difficulties experienced in the past 6 months. For the purposes of synthesis, these three measures (financial difficulties experienced, considering dropping out due to financial issues, and the IFS) were all considered to be measures of financial difficulties. Three studies asked about debt worry (also called debt stress), and two studies asked about financial concern; these were also grouped.

The majority of studies (8 of 11) used questionnaires commonly used for measuring general mental health in the general population, such as the General Health Questionnaire (GHQ)^18^ (four studies) and the General Population version of the Clinical Outcomes in Routine Evaluation Questionnaire^19^ (CORE-GP) (three studies). Two of these studies also used specific anxiety and depression measures. The remaining three studies used only measures of anxiety and depression (Hospital Anxiety and Depression Scale (HADS), psychosis Prodromal Questionnaire-Brief (PQB) (Version) and disordered eating behaviours Eating Attitudes Test-26 (EAT-26), respectively). Additionally, two studies measured examination results and one asked about help seeking via general practitioner and satisfaction around help seeking. The characteristics and key findings of each study can be found in table 1.

### Debt and mental health

Seven studies investigated the relationship between amount of debt and mental health. Of these, three found an association between more debt and poorer mental health, and one found an association between less debt and poorer mental health. The remaining three studies reported no association.

Cooke *et al* 11 found no association between anticipated debt and mental health among final year students from one UK university (n=2146). Similarly, Richardson *et al* 8 found no difference longitudinally in general mental health, depression or anxiety between groups with different tuition fee debt amounts (n=390). The only study that looked at psychosis also found no evidence that debt was cross-sectionally associated with psychosis risk when gender, age and ethnicity were controlled for.^20^

One small study^16^ (n=89) found that higher debt was associated with poorer mental health and more financial concern. Two other cross-sectional studies^21 22^ (n=103 and n=360) used structural equation modelling to estimate that larger debt was associated with worse mental health through longer hours worked or a higher likelihood of considering abandoning studies.

On the other hand, Ross *et al* 7 found in one university (n=334) that students with worse mental health had less debt, though students who reported that worrying about money affects their academic performance had significantly higher debt than those who did not. This study also found that there was no association between debt amount and academic performance (examination results).

### Financial difficulties and mental health

All seven studies investigating the relationship between experiencing financial difficulties and poorer mental health found some association between the two, though longitudinal and bidirectional findings were mixed.

All three studies by Roberts *et al* 6 21 22 used cross-sectional data from two UK universities (n=103, n=360, and n=408) and reported that difficulty paying bills was associated with poorer mental health. All three also reported that participants who had considered abandoning their studies due to financial reasons had poorer mental health than those who had not.

Andrews and Wilding^2^ used longitudinal data collected across two time points (n=351), and found that experiencing financial difficulties during university (such as being unable to afford essentials) was associated with the development of depression midcourse, but not anxiety. This study also reported a relationship between more financial difficulties and worse examination performance at the end of second year, a relationship that was mediated by the midcourse depression scores. Another longitudinal study^14^ found that those who reported experiencing financial difficulties at baseline (according to the IFS) were also more likely to report anxiety, depression and stress cross-sectionally, and anxiety 2 months later. Those who indicated at baseline that they had considered abandoning their studies for financial reasons had higher depression scores 6 and 8 months later. None of the mental health measures at baseline were associated with IFS scores 2 months later, though worse general mental health at baseline was associated with more financial difficulties 4 months later.

The only study that looked at psychosis outcomes observed no relationship between considering abandoning studies for financial reasons and psychosis symptoms.^20^ However, this study found that higher IFS scores at baseline were associated with both increased mental distress and psychosis symptoms after 4 months, and distress after 6 months. Conversely, higher psychosis symptom scores at baseline were not associated with higher IFS scores at either follow-up points. Lastly, one study concluded that experiencing financial difficulties (higher IFS score) at baseline was associated with more severe eating attitudes long term (at 8 and 12 months), but only in women.^23^ Severe eating attitudes at baseline were associated with higher IFS scores at the 4 month time point only (not at 8 or 12 months).

### Debt worry/financial concern and mental health

Four out of five studies found an association between an indicator of financial concern or debt worry and poorer mental health (table 1). The fifth study (n=408) found that students’ reported stress about debt was not associated with their psychosis risk.^20^

Cooke *et al* 11 collected data from undergraduates at one university (n=1391) every year for 3 years, and observed an association between more financial concern (measured by one question) and worse mental health at every time point. This study also found that, among third year students, debt worry was associated with worse general mental health. When students were split into groups according to ‘no debt worry’, ‘low debt worry’ and ‘high debt worry’, there was some evidence for differences between the ‘low’ and ‘high’ groups in outcomes such as feeling more unhappy and irritable and less able to cope, but fewer differences between the ‘no’ and ‘high’ groups.

A second longitudinal study^14^ demonstrated that greater subjective stress about debt at baseline was associated with depression cross-sectionally, and with greater anxiety, stress and poorer general mental health cross-sectionally and at the longest follow-up point (around 8 months).

One particularly small study^16^ (n=89) found that those with more financial concern (more agreement with statements such as ‘I worry about my financial situation’) had worse general mental health, but were not more likely to indicate that their mental health affects their everyday functioning.

Finally, Ross *et al* 7 (n=334) identified that students with poorer mental health were more likely to report that worrying about money affects their academic performance. Additionally, students who indicated that they thought that worrying about money affected their performance on average ranked lower in academic performance than students who did not indicate this.

## Discussion

### Summary of evidence

In summary, this review identified 11 papers (seemingly from 6 separate studies) examining the associations between indicators of financial stress and mental health among higher education students in the UK. Findings differed by financial stress indicator. There was little evidence that debt was associated with mental health—the only studies that reported an association were either very small or suggested that debt impacted on mental health through the experience of financial difficulties. Financial difficulties were cross-sectionally associated with worse general mental health, and increased symptoms of anxiety and depression. However, longitudinal findings were mixed and generally differed by follow-up time point and measure. There was some very limited evidence for bidirectional relationships between financial difficulties and both general mental health and severe eating attitudes, but not anxiety, depression and psychosis symptoms. Evidence was more consistent for a relationship between mental health and the subjective measures of debt worry/financial concern. Four studies reported a cross-sectional relationship between these indicators and mental health outcomes including general mental health, anxiety and depression. However, only two studies collected longitudinal data for this indicator.

Taken together, we believe that the strength of the identified evidence is weak, such that it is unclear how robust the associations are between financial stress and mental health among UK higher education students. While evidence indicates that more subjective measures of financial stress may be associated with worse mental health, it is unclear whether the reported associations reflect causal relationships, whether these act in either or both directions and whether they have long-term implications.

### Limitations of the available evidence

The studies included were typically very small in size, ranging from 89 to 2146 (median=408), and many were conducted at just one UK university. This field may be particularly vulnerable to publication bias as a result, with small studies reporting null findings less likely to get published. It is also unclear whether these findings generalise to the UK higher education student population as a whole, particularly as key factors such as available support differ considerably by university.

In many papers, key information such as survey dates and analytical strategies was not provided, which made appraisal challenging. Response rates were frequently not reported,^6 14 20 23^ which means that the possibility of selection bias could not be appraised—those experiencing financial difficulties and mental health problems may be less likely to respond to surveys, potentially leading to an underestimation of the association between these variables. Additionally, where multiple papers with the same lead author are included, they appear to be reporting on findings from the same study, but without additional details we cannot be completely certain.

Finally, studies were by design limited in the extent to which they could make causal claims regarding the relationship between financial stress and mental health. Cross-sectional studies are unable to demonstrate the direction of causality, and this is often still not possible in the longitudinal analyses where follow-up length was limited.

The associations reported may also be confounded by other variables such as socioeconomic factors, and many studies did not account for these in their analyses.^6 11 23^ Even where adjustment is made, residual confounding or confounding due to omitted variables remains a potential explanation for the reported associations. As such, empirical strategies that rely on alternative assumptions for causal inference are likely to be useful in future. Changes across time and place in exposure to debt (eg, the 2012 tuition fee rises from £3000 to £9000 in England; yet there are no fees in Scotland) could provide potentially exogenous (unconfounded) sources of variation with which to examine the causal effects of debt on mental health outcomes. Such a design would require comparable data across time and/or place, and would likely require large sample sizes to be sufficiently powered (eg, to test interaction terms in a difference-in-difference design).^24^

### Limitations of this review

There are several limitations of this review. Rapid reviews are increasingly used in evidence synthesis,^25^ and may be a more efficient alternative to full systematic reviews which typically take substantially longer (eg, 24 months vs 2) and may yield similar findings,^15^ particularly if multiple databases are searched.^26^ However, there remains more uncertainty in the conclusions drawn in a rapid versus full systematic review, and the extent of this uncertainty is specific to the research question and which aspect of the review process was expedited. In this review, while multiple search engines were used, and we additionally manually searched for relevant papers, it is possible that some relevant evidence was missed, since only one author screened papers. A full systematic review could therefore be warranted. This review was also limited to studies conducted in the UK. Understanding how this association differs across countries would be useful in order to better understand the relative importance of financial stress with regards to student mental health, and identify support systems and policies that may alleviate links between them. As such, future work could consider a broad range of countries, while examining potential reasons for cross-national similarities and differences in the association between financial stress and mental health.

## Conclusions

In summary, while evidence in this field is still very limited, it appears that financial stress, as indicated by students’ financial concern, may be associated with mental health outcomes—potentially more so than the amount of debt accrued. Given ongoing concerns regarding student mental health, further research should use robust methods and a range of financial stress measures to establish whether there are long-term causal relationships between financial stress and mental health.

**What is already known on this subject**

- Increases in student debt in recent decades have coincided with increased rates of mental health problems and deaths by suicide among higher education students. However, it is unclear whether financial stress is associated with mental health outcomes among this population, nor which aspects of increased financial stress may be important.

**What this study adds**

- In a rapid review of links between student financial stress and mental health, we found that some indicators of financial stress (such as financial concern and experience of financial difficulties) were associated with worse mental health, but amount of debt was not. However, the strength of evidence was judged to be weak. Given ongoing concerns regarding high rates of student mental health problems, further research is needed to establish whether there are long-term causal relationships between financial stress and mental health, with a focus on the various types of financial stress students face. Additionally, understanding how the association between financial stress and mental health differs between countries would also be useful in order to identify beneficial support systems and policies.
